# A Machine Learning Approach for the Detection of QRS Complexes in Electrocardiogram (ECG) Using Discrete Wavelet Transform (DWT) Algorithm

**DOI:** 10.1155/2022/9023478

**Published:** 2022-04-28

**Authors:** Ali Rizwan, P Priyanga, Emad H. Abualsauod, Syed Nasrullah Zafrullah, Suhail H. Serbaya, Awal Halifa

**Affiliations:** ^1^Department of Industrial Engineering, Faculty of Engineering, King Abdulaziz University, Jeddah 21589, Saudi Arabia; ^2^Assistant Professor, Department of CSE, RV Institute of Technology and Management, Bengaluru, India; ^3^Department of Industrial Engineering, College of Engineering, Taibah University, Madina Almonawara 41411, Saudi Arabia; ^4^Department of Information Systems, College of Computer Engineering & Sciences, Prince Sattam Bin Abdulaziz University, Al-Kharj 11942, Saudi Arabia; ^5^Kwame Nkrumah University of Science and Technology, Kumasi, Ghana

## Abstract

This study describes a modified approach for the detection of cardiac abnormalities and QRS complexes using machine learning and support vector machine (SVM) classifiers. The suggested technique overtakes prevailing approaches in terms of both sensitivity and specificity, with 0.45 percent detection error rate for cardiac irregularities. Moreover, the vector machine classifiers validated the proposed method's superiority by accurately categorising four ECG beat types: normal, LBBBs, RBBBs, and Paced beat. The technique had 96.67 percent accuracy in MLP-BP and 98.39 percent accuracy in support of vector machine classifiers. The results imply that the SVM classifier can play an important role in the analysis of cardiac abnormalities. Furthermore, the SVM classifier also categorises ECG beats using DWT characteristics collected from ECG signals.

## 1. Introduction

One of the foremost reasons of death in the world is heart disease and heart stroke. Early detection of arrhythmia situations via ECG signal analysis could improve the identification of risk factors for cardiac arrest. Baseline drifts and power line interference along with physiological abnormalities distort the ECG signal [1]. ECG signal noises can be eliminated using a variety of approaches. Mathematical morphology (MM) [2], empirical mode decomposition (EMD) [3], adaptive filtering (AF) [4], weighted averaging filter (WAF) [5], and independent component analysis (ICA) [6] are some of the most commonly used methods. Electromyography (EMG) noise can be eliminated by using adaptive wavelet wiener filtering of ECG signals [7]. The characteristics of an ECG signal can be analysed statistically [8] and retrieved in time and frequency domains [9, 10]. An accurate reading of the QRS complex is essential to ECG analysis because it contains the majority of the heart's electrical action [11]. Preprocessing techniques such as filtering and smoothing are used to reduce P and *T* waves and noise [12]. The uncovering of the QRS onset and offset facts, which begin with the R-peaks, is the first step in computing the QRS duration. ECG signal analysis can be improved by locating the correct fiducial point [13, 14]. Digital filters and nonlinear transforms are utilised to excerpt the feature constituents of the QRS signal [15, 16]. The modulus maxima were found using a multiscale QRS indicator employing discrete wavelet transform (DWT). There are numerous signal processing applications that use the DWT algorithm. Constants that indicate the adequate evidence of the real data are used to divide DWT signals into different coarseness levels [17, 18] [19–21]. Using WT's multiscale feature, it is possible to detect the QRS complex and P wave and *T* waves with 99.5% accuracy even in the occurrence of baseline drift and noise. Martinez et al. [18, 22] proposed a dyadic wavelet-based method for locating the maximum positive and minimum negative peaks. To evaluate the ECG signal's QRS complex, P wave, and *T* waves, a multiresolution wavelet transform system with optimal coefficients was applied. There is 99.9% accurate recognition rate for R-peak and a base accurateness of 97.6%, 96.65%, and 98.85% of heart rate for P wave, *T* wave, and QRS complex correspondingly, in these measurements [21]. Rescaled wavelet coefficients were utilised to enhance the QRS complex and lessen its impact on other peaks [23]. QRS can be detected with a classification based on morphology that has a 99.91 percent average sensitivity and a 99.72 percent positive predictivity [24]. When used with adaptive thresholding to detect the peaks and waves of ECG signals, multiresolution WT showed 99.8 percent sensitivity and positive predictivity in the MIT-BIH arrhythmia database and 99.84 percent in the PTB database [25]. The deconstructed signals' power spectra were utilised to generate a multiresolution wavelet transform, which was then used to determine detail coefficients for the QRS complex's parameters 1 and 2 [26]. An error rate of 0.34 percent was found in the evaluation, which exhibits the best detection performance with 99.87 percent global sensitivity and 99.79 percent positive predictivity [26]. Wavelet coefficients and wavelet coefficient energy were used for detection in the real-time QRS complex detector developed by Junior et al. [22] in 2016. The algorithm's detection rate for QRS complexes was 99.32 percent. The identification and classification of cardiac arrhythmias are the primary goals of this study. The classification of cardiac arrhythmia has been the subject of much debate in the medical literature. SVMs (support vector machines) [27] and ANNs (artificial neural networks) [28] are two such approaches. Profligate and precise categorization of cardiac rhythms has been achieved using an OPF classifier based on supervised graph pattern recognition [29].

Pathological situations alter ECG morphologies; therefore, designing a robust algorithm to accurately detect QRS complexes is a challenge [11]. The multiresolution wavelet transform and amplitude thresholding are used in this study to offer an upgraded approach for detecting QRS complex structure and R-peaks. Neural networks and support vector machines (SVM) were used to identify four cardiac abnormalities: normal (N), left bundle branch block (LBBB) and right bundle branch block (RBBB), and paced beats (P) [30]. An ECG database of 48 documented ECG data from MIT-BIH arrhythmias was used to estimate the enactment of the proposed procedure for sensitivity, specificity, and accuracy as the parameter.

Motivation: QRS complex is the most notable feature and can be used to extract further clinical information from ECG signals, such as RR interval, QT interval, and PR interval, among other things [2]. Detecting QRS on an ECG is, therefore, essential for health assessment based on the ECG. The discrete wavelet transform (DWT) techniques are well established in signal processing across a wide range of scientific and industrial fields [7]. DWT is regularly employed to answer and cure more complex problems since it gives both octave-scale frequency and spatial time of the investigated signal.

## 2. QRS Detection Approaches

**Figure 1:**
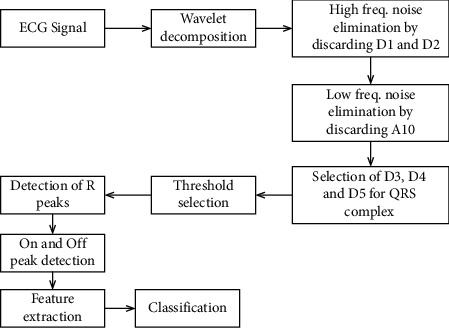
Block diagram of the DWT-based ECG beat detection algorithm.

MATLAB was used to transform the ECG signals from the MIT-BIH arrhythmia database, which had sampling frequency, set to 360 Hz (.mat). A 10 mV range of 11 bit resolution ECG signals is used to store 48 recordings of 30 minute duration each.  Figure 1 shows a block diagram of the proposed algorithm for detecting the QRS complex in ECG signals [31]. Denoising, QRS detection, R-peak identification, on and off peaks, and morphological parameter extraction are all part of this process. It was evaluated on the first channel's ECG signals using MATLAB 2018 version of the technique. Approximately 1.33 seconds of each 30 minute ECG dataset was required for processing [32].

### 2.1. Discrete Wavelet Transform (DWT)

The Fast Fourier Transform (FFT) is an important tool for signal processing applications that analyse stationary signals. A nonstationary signal does not match the performance requirements of this device [33]. While you are seeking for a means to analyse temporal frequency, you can utilize the short time Fourier transform (STFT). Time frequency resolution is the primary challenge with STFT. STFT's disadvantage can be rectified by employing the wavelet transform, which provides good temporal and frequency resolution. Using the wavelet transform to evaluate nonstationary signals such as ECG is an efficient method because of its ability to localise time and frequency [34]. Biomedical signal processing makes extensive use of DWT because of its lower computational complexity than that of FFT. ECG signal decomposition is also used to remove noise and enhance ECG signal components by scaling the ECG signal. An w(t) signal's wavelet transform is defined by

(1) $$\begin{array}{c} L\left(d,l\right)=\frac{1}{\sqrt{m}}\int_{-\infty }^{+\infty }w\left(t\right).\varphi^{*}\left(\frac{t-l}{d}\right)\text{d}t. \end{array}$$

Complex conjugate of wavelet transform *φ*^*∗*^ (t) and dilation and location parameters of the wavelet, respectively, are *d* and *l* in this equation. The orthonormal wavelet basis function is used in DWT's dyadic grid. If you use a discretized grid, such as the d-l grid, to calculate the transform integral, you will get the same result. One way to express the DWT function is as follows [35]:

(2) $$\begin{array}{c} \varphi \left(m,n\right)=\frac{1}{\sqrt{p^{m}}}\int_{-\infty }^{+\infty }w\left(t\right).\varphi^{*}\left(\frac{t-nqp^{m}}{p^{l}}\right)\text{d}t. \end{array}$$

Dilation and translation are denoted by *m* and *n*, respectively. The default settings for the dilation (*p* > 1) and location (*q* > 0) parameters are *m* and *b*, respectively.

When trying to identify the QRS complex in an ECG waveform, wavelet selection is critical. There is no way to select a wavelet that is accurate [35]. The wavelet is chosen based on the information included in the processed signal. The following wavelets are readily available: Daubuchies, Haar, Biorthogonal, Symlets, Morlets, Mexican Hats, Mayers, and Coiflets.

#### 2.1.1. Preprocessing

The most crucial component in signal decomposition is to select a wavelet type that closely counterparts the morphology of the data under deliberation [36]. To enhance the QRS complex shape, noninformative frequency constituents were decomposed with multiresolution wavelet transform utilising db6 in the preprocessing step. To improve the signal, noise with equivalent detail coefficients at D1, D2, and A10 is removed from the high frequency ranges at D1 and D2.

#### 2.1.2. Detection of R-Peaks

**Figure 2:**
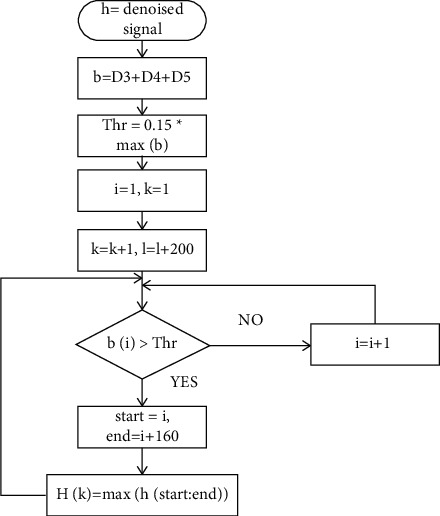
R-peak detection flow diagram.

R-wave locations are defined by the denoised ECG signal's greatest amplitude.  Figure 2 depicts the detection process for Rloc. In order to locate the window of 160 ms around the QRS area, a pragmatic verge of 15% of the maximal amplitude addition of D3, D4, and D5 was carefully determined. To find amplitudes greater than the predefined edge level, the ECG beats are denoised. A signal's R-peak placements are determined by its highest amplitude [35]. Ventricular depolarization cannot be detected during the refractory period, which is 200 ms. In an array of Rloc, the identified supreme peaks are kept as “R-peaks.”

## 3. Classification Methods

We have studied various classification methods. Few of them are mentioned below.

### 3.1. Multilayer Perceptron Neural Network (MLP)

In ECG signal investigation, the neural network classifier is the most commonly utilised [1]. The neural network topology known as “multilayer perception” (or “MLP”) is widely used. Using the backpropagation algorithm, MLPs are trained by allowing mistakes to circulate over the network and allowing adaption of the secreted nodes [37]. Simply stated, its function is

(3) $$\begin{array}{c} B=\sum_{i=1}^{d}A_{i}X_{ij}, \end{array}$$

where *A*_*i*_ is input as *A* =  [*a*_1_, *a*_2_ … …*a*_*n*_]^*T*^ and *X*_*ij*_ are set of weights *X*=[*x*_1_, *x*_2_ … …*x*_*n*_]^*T*^.

Moreover, error can be calculated as

(4) $$\begin{array}{c} e_{i}\left(n\right)=d_{i}\left(n\right)-B_{i}\left(n\right). \end{array}$$

When learning with gradient descent, the input and error values at each weight in the network are corrected, i.e.,

(5) $$\begin{array}{c} x_{ij}\left(n+1\right)=x_{ij}\left(n\right)+\partial \delta_{i}\left(n\right)a_{i}\left(n\right). \end{array}$$

**Figure 3:**
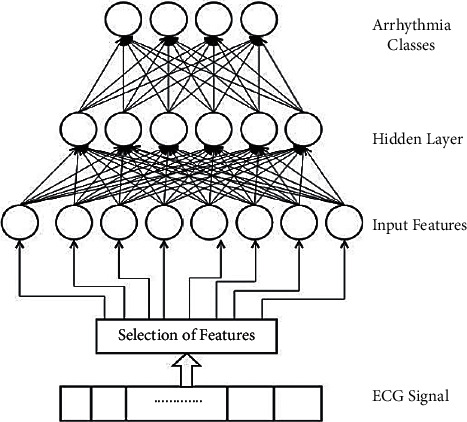
MLP neural network architecture.

Figure 3 depicts the investigational design of MLP, which is created with eight input topographies in the input layer, one secreted layer, and four modules in the output layers to categorise ECG rhythms.

### 3.2. Support Vector Machine (SVM)

This very nonlinear network topology uses the notion of structural risk minimization to accurately classify previously unknown patterns [38]. Support vector machines lower structural risk, whereas empirical risk has increased. SVM maximises the expanse between the configurations and the hyperplane. It optimises the class separation boundary in order to keep the distance between a feature and a class 29 separating hyperplane at its largest simultaneous value. Kernels for training include quadratic, polynomial, and radial basis functions [23].

**Figure 4:**
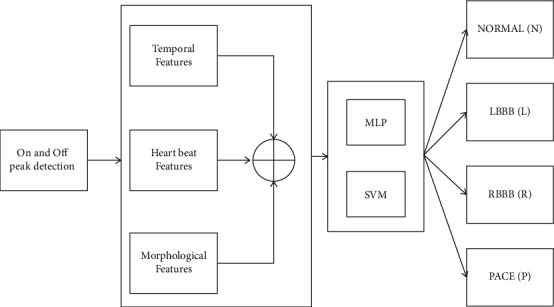
ECG beats' classification process block diagram.

The fiducial point interludes of an ECG in a cardiac sequence are used to classify an ECG signal automatically. Each heartbeat had its own set of intervals and ECG morphological traits.  Table 1 lists the characteristics of a single cardiac cycle. In Figure 4, the ECG classification approaches are depicted as a block diagram. Using the ECG preprocessed signal, the proposed classification technique extracts temporal, R-R interval, and morphological information [2]. The NN classifier uses a combination of variables to categorise various cardiac problems (Paced beats, Normal, RBBBs, and LBBBs).

## 4. Evaluation Measure and Results

Filters are employed in a different ways, liable on the explicit application. In order to pick the best technique and develop new methodologies, performance measures are used [9]. Metrics such as accuracy, signal-to-noise ratio (SNR), mean square error (MSE), specificity, and sensitivity are deliberated by comparing the enactment of prevailing and new methods.

### 4.1. Accuracy

Metrics count the number of times a forecast was correct out of all the possible outcomes:

(6) $$\begin{array}{c} \text{Accuracy}=\frac{\text{TP}+\text{TN}}{\text{TP}+\text{FP}+\text{TN}+\text{FN}}, \end{array}$$

where for TP : True Positive, correct value is detected as correct, for TN : True Negative, incorrect value is detected as correct, for FP : False Positive, correct value is detected as incorrect, and for FN : False Negative, incorrect value is detected as incorrect.

### 4.2. Sensitivity

Measure the percentage of positive patterns which is accurately categorised. The percentage of incorrectly categorised negative patterns is what we refer to as sensitivity:

(7) $$\begin{array}{c} \text{Sensitivity}=\frac{\text{TP}}{\text{TP}+\text{FN}}. \end{array}$$

TP and FN are mentioned in equation (7).

### 4.3. Specificity

The difference between the projected and desired solutions is what we mean by “specificity:”

(8) $$\begin{array}{c} \text{Specificity}=\frac{\text{TN}}{\text{TP}+\text{FP}}. \end{array}$$

TN, TP, and FP are mentioned in equation (8).

### 4.4. Detection Error Rate (DER)

(9) $$\begin{array}{c} \text{DER}=\frac{\text{FP}+\text{FN}}{\text{TP}}X\;100\%. \end{array}$$

### 4.5. Positive Predictability (+P)

The algorithm's ability to distinguish between real and fake beats is evaluated using positive predictability:

(10) $$\begin{array}{c} +p=\frac{\text{TP}}{\text{TP}+\text{FP}}X\;100\%. \end{array}$$

## 5. Results and Discussion

The MIT-BIH arrhythmia database was applied to test the suggested R-peak and QRS complex recognition methods. Excellence, waves, QRS complex, and uneven cardiac beats of the recorded ECG signals are all within acceptable limits.

### 5.1. Detection of the R-Peak

**Figure 5:**
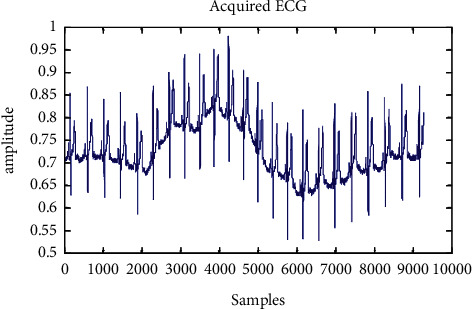
Novel assimilated abnormal ECG signal.

**Figure 7:**
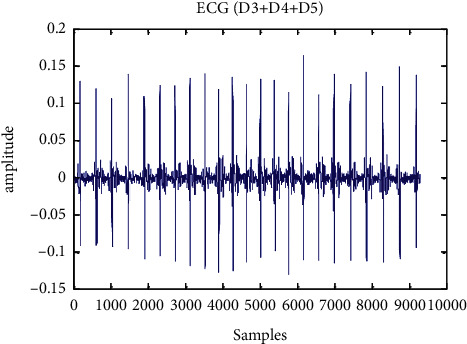
Recognition of (sum of D3, D4, and D5) particular region of ECG signal.

**Figure 8:**
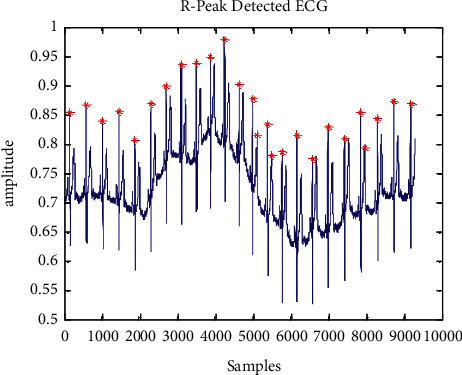
R-peak identified of record no. 117 of ECG signal.

An aberrant ECG signal of 117 records is displayed in Figure 5 to Figure 7 after a wavelet transform and baseline wander correction.  Table 2 summarises the consequences of all 24 accounts in the MIT-BIH arrhythmia database, which are shown in Figure 8 as simulations of R-peak detection.

### 5.2. Onset and Offset Recognition of ECG Peaks

**Figure 9:**
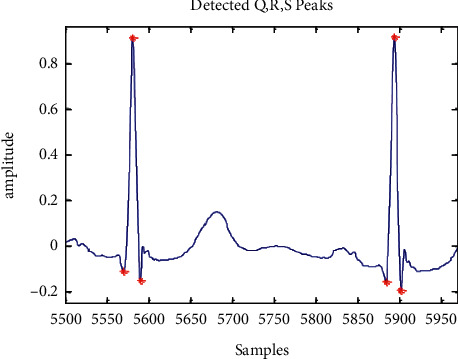
QRS complex detection simulation result.

**Figure 10:**
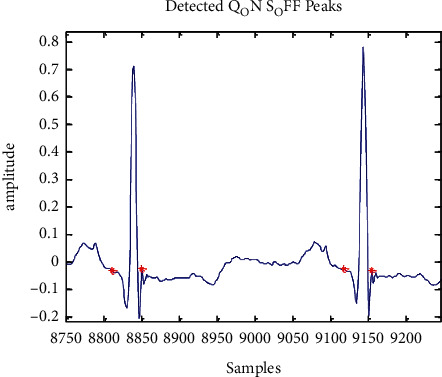
*Q* On and *S* Off peak detection simulation result.

**Figure 11:**
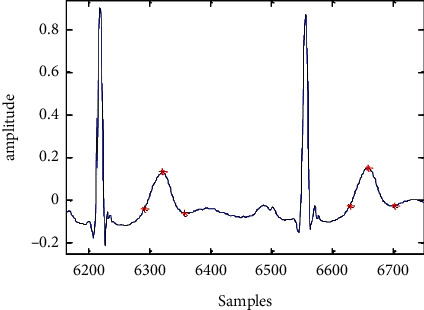
*T* On and *T* Off peak detection simulation result.

The QRS complex can be found once the R-peak and *Q* and S points have been located exactly [25]. In order to locate the Q-peak (Qloc), 30 samples are examined for a negative maximum on the leftward side of the fiducial point (Rloc). Fifty samples are selected from the right-side fiducial area in order to indicate Sloc.  Figure 9 and 10 show the minimum slopes for the *Q* and S positions, which are kept in a collection of Q-index and S-index. An examination is launched by picking 20 samples to the leftward of Qloc and to the rightward of Sloc. An Rloc range of 30 to 160 was selected for the T-peak localization, and the largest value within this range is considered Tloc. Thirty five and fifty samples are selected in a search procedure, which is a standard sample set for a regular *T* wave duration. Tloc's absolute minima on either side are depicted in Figure 11 as *T* On and *T* Off, respectively.

## 6. Conclusion

Methods for extracting features from the multiresolution wavelet transform (MLP-BP) and categorising for cardiac irregularities by machine learning (SVM) are presented in this study. Through a recognition fault rate of 0.45 percent, the suggested method outperforms existing methods in the recognition of QRS complexes in terms of specificity and sensitivity, compared to previously published data. In terms of classification accuracy, the classifiers agreed that our proposed strategy was preferable for categorising four different types of ECG rhythms: LBBBs, Normal, RBBBs, and Paced beats. In the classifiers, such as MLP-BP and support vector machine, the method attained a mediocre classification accuracy of 96.57 and 98.59 percent, respectively. Support vector machine (SVM) classifiers may play a noteworthy part in the clarification of information in the identification of heart issues. According to the findings of this study, categorising ECG rhythms using DWT-based topographies collected from ECG data was found to be feasible using the SVM classifier. We found that the SVM classifier can categorise ECG beats [39] using DWT characteristics taken from ECG signals.

In future, we will try to design a method which will applicable for real-time data. Moreover, the method will be applicable on big data too.

## Figures and Tables

**Figure 6:**
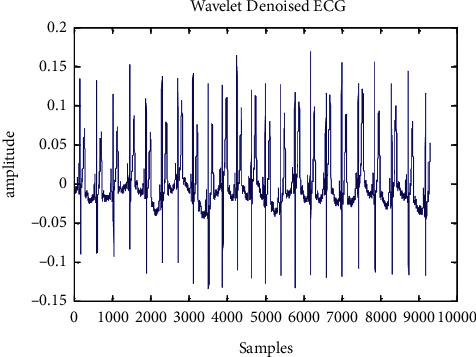
Wavelet filtered ECG signal of record 117.

**Table 1:** ECG features extracted.

Group	Temporal	Heart beat features	Morphology
Features	R-R intervals	Amplitudes of Q, R, S and T, QRS extent	Q-T interlude and S-T interlude

**Table 2:** QRS complex detection.

Record no.	TB	DB	TP	FP	FN	% Se	%Pp	% DER
100	2273	2273	2273	0	0	100	100	0
100	2273	2273	2273	0	0	100	100	0
101	1865	1866	1863	3	6	99.89	99.83	0.26
102	2187	2187	2181	0	0	99.72	99.72	0.54
103	2084	2084	2084	6	2		100	0
104	2222	2260	2224	2	3	100	98.4	1.83
105	2572	2579	2569	12	5	99.77	99.61	0.5
106	2026	2025	2023	34	4	99.8	99.9	0.29
107	2136	2150	2134	3	3	99.88	99.25	0.88
108	1763	1850	1755	18	4	99.54	94.86	5.84
109	2532	2519	2528	93	8	99.85	99.96	0.19
111	1763	1850	2124	1	0	100	100	0
112	1987	1988	2539	1	0	99.23	99.96	0.03
113	1518	1518	1759	0	1	100	99.94	0.05
114	1619	1619	1879	1	0	99.12	99.73	0.26
115	2477	2477	1953	4	0	100	100	0
116	2325	2206	2391	2	2	100	99.87	0.99
117	2251	1879	1535	5	0	99.94	99.67	0.32
118	2345	1468	2278	3	3	100	99.79	0.08
119	1589	1654	1984	5	0	100	99.91	0.35
121	2145	2345	2476	3	0	100	99.83	0.21
122	1981	1548	1518	4	1	99.84	100	0
123	1245	1680	1852	0	0	99.93	99.93	0
124	1546	1897	1628	2	1	100	100	0.12

## Data Availability

The data that support the findings of this study are available on request from the corresponding author.
